# *QuickStats:* Percentage* of Office-Based Physicians Using Telemedicine Technology,^†^ by Specialty^§^ — United States, 2019 and 2021

**DOI:** 10.15585/mmwr.mm7149a6

**Published:** 2022-12-09

**Figure:**
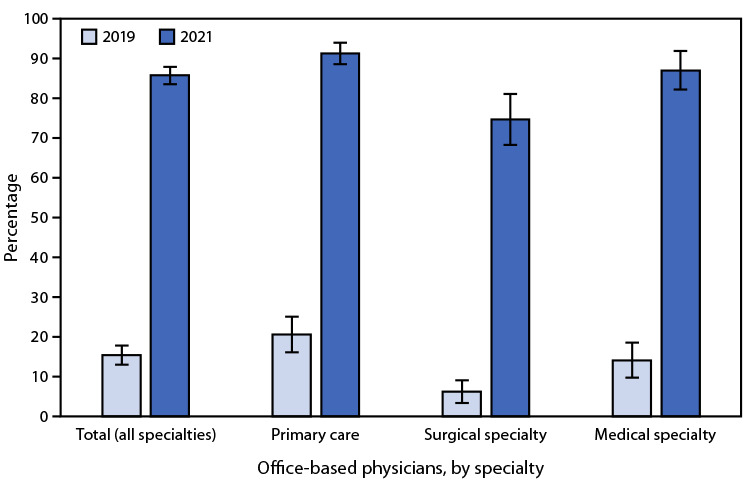
From 2019 to 2021, the use of telemedicine technology increased for office-based physicians from 15.4% to 85.9%. In both 2019 and 2021, the use of telemedicine technology was higher among primary care physicians and medical specialty physicians than it was among surgical specialty physicians. In 2021, 91.4% of primary care physicians, 87.2% of medical specialty physicians, and 74.8% of surgical specialty physicians used telemedicine technology.

**Sources:** National Center for Health Statistics, National Electronic Health Records Surveys, 2019 and 2021. https://www.cdc.gov/nchs/nehrs/about.htm

